# 

**Published:** 1805-01-01

**Authors:** 


CONDUCTED BY

T. BRADLEY, M.D.
R. BATTY, M.D.
AND
A. A. NOEHDEN, M.D.

S.G.O.

VOL. XIII.

FROM JANUARY TO JUNE, 1805.

S.G.O. LIBRARY, Washington, D.C. 5119

*LONDON:*

PRINTED FOR RICHARD PHILLIPS, NO. 6, BRIDGE-STREET, BLACKFRIARS.

[By William Thorne, Red Lion Court, Fleet Street.]

W
1
ME 182

### To CORRESPONDENTS.

Communications are received from Drs. Domeyer, Hall, King, Male, Trotter, and Weatherhouse; Messrs. Hume, Scott, Taynton, and Wilkinson.

